# Respiratory drive heterogeneity associated with systemic inflammation and vascular permeability in acute respiratory distress syndrome

**DOI:** 10.1186/s13054-024-04920-4

**Published:** 2024-04-23

**Authors:** Elias Baedorf-Kassis, Michael Murn, Amy L. Dzierba, Alexis L. Serra, Ivan Garcia, Emily Minus, Clarissa Padilla, Todd Sarge, Valerie M. Goodspeed, Michael A. Matthay, Michelle N. Gong, Deborah Cook, Stephen H. Loring, Daniel Talmor, Jeremy R. Beitler, for the EPVent-2 Study Group, Daniel Talmor, Todd Sarge, Valerie Goodspeed, Emily Fish, Sayuri Jinadasa, Ray Ritz, Joseph Previtera, Michelle N. Gong, Lawrence Lee, Jeremy R. Beitler, Deborah Cook, France Clarke, Tom Piraino, Joseph Levitt, Rosemary Vojnik, Pauline Park, Kristin Brierley, Carl Haas, Andrew Weirauch, Eddy Fan, Andrea Matte, R. Scott Harris, Mamary Kone, Stephen Heard, Karen Longtine, Franćois Lellouche, Pierre-Alexandre Bouchard, Lewis Rubinson, Jennifer McGrain, Donald E. G. Griesdale, Denise Foster, Richard Oeckler, Amy Amsbaugh, Edgar Jimenez, Valerie Danesh

**Affiliations:** 1 Division of Pulmonary and Critical Care Medicine, Beth Israel Deaconess Medical Center, Harvard Medical School, Boston, MA USA; 2 Columbia Respiratory Critical Care Trials Group, Columbia University College of Physicians and Surgeons, and New York-Presbyterian Hospital, 622 West 168th Street, New York, NY 10032 USA; 3 Center for Acute Respiratory Failure, New York-Presbyterian Hospital, New York, NY USA; 4 Department of Pharmacy, New York-Presbyterian Hospital, New York, NY USA; 5 Departments of Medicine and Anesthesia, University of California San Francisco, San Francisco, CA USA; 6 Department of Anesthesia, Critical Care, and Pain Medicine, Beth Israel Deaconess Medical Center, Harvard Medical School, Boston, MA USA; 7 Department of Critical Care Medicine, Montefiore Medical Center and Albert Einstein College of Medicine, Bronx, NY USA; 8 St. Joseph’s Hospital and McMaster University, Hamilton, ON Canada

**Keywords:** Acute respiratory distress syndrome, Mechanical ventilation, Hypnotics and sedatives, Respiratory mechanics, Work of breathing

## Abstract

**Background:**

In acute respiratory distress syndrome (ARDS), respiratory drive often differs among patients with similar clinical characteristics. Readily observable factors like acid–base state, oxygenation, mechanics, and sedation depth do not fully explain drive heterogeneity. This study evaluated the relationship of systemic inflammation and vascular permeability markers with respiratory drive and clinical outcomes in ARDS.

**Methods:**

ARDS patients enrolled in the multicenter EPVent-2 trial with requisite data and plasma biomarkers were included. Neuromuscular blockade recipients were excluded. Respiratory drive was measured as P_ES_0.1, the change in esophageal pressure during the first 0.1 s of inspiratory effort. Plasma angiopoietin-2, interleukin-6, and interleukin-8 were measured concomitantly, and 60-day clinical outcomes evaluated.

**Results:**

54.8% of 124 included patients had detectable respiratory drive (P_ES_0.1 range of 0–5.1 cm H_2_O). Angiopoietin-2 and interleukin-8, but not interleukin-6, were associated with respiratory drive independently of acid–base, oxygenation, respiratory mechanics, and sedation depth. Sedation depth was not significantly associated with P_ES_0.1 in an unadjusted model, or after adjusting for mechanics and chemoreceptor input. However, upon adding angiopoietin-2, interleukin-6, or interleukin-8 to models, lighter sedation was significantly associated with higher P_ES_0.1. Risk of death was less with moderate drive (P_ES_0.1 of 0.5–2.9 cm H_2_O) compared to either lower drive (hazard ratio 1.58, 95% CI 0.82–3.05) or higher drive (2.63, 95% CI 1.21–5.70) (*p* = 0.049).

**Conclusions:**

Among patients with ARDS, systemic inflammatory and vascular permeability markers were independently associated with higher respiratory drive. The heterogeneous response of respiratory drive to varying sedation depth may be explained in part by differences in inflammation and vascular permeability.

**Supplementary Information:**

The online version contains supplementary material available at 10.1186/s13054-024-04920-4.

## Background

Respiratory drive can vary considerably between patients receiving mechanical ventilation for acute respiratory failure [1]. Preserving some level of respiratory drive seems important to preventing diaphragm disuse atrophy and functional respiratory muscle weakness, which otherwise may impede liberation from the ventilator [2, 3]. However, high drive might predispose to lung and diaphragm injury through dyssynchronous patient-ventilator interactions, high tidal volumes, and excessive diaphragm load [4, 5].

Critical illness is accompanied by a multitude of physiological perturbations that can influence respiratory drive [6–8]. These factors may include chemoreceptor input from acid–base disturbances, hyper-/hypocapnia, and hyper-/hypoxemia; mechanoreceptor input resulting from pulmonary atelectasis, consolidation, and edema; and suprapontine input from depressed consciousness, anxiety, pain, and accompanying analgesics/sedatives [8]. To an extent, each of these factors is observable, and often routinely monitored, in clinical care.

We have found that substantial heterogeneity in respiratory drive exists among adult patients with acute respiratory failure even after accounting for these clinically observable factors [1]. Inflammation and vascular permeability, though not as readily quantifiable clinically, may also influence respiratory drive [9–12]. Both systemic inflammation and vascular permeability are thought to be potential mediators of lung injury, multiorgan dysfunction, and mortality in ARDS [13]. While hyperinflammatory states and endothelial barrier dysfunction are common in critical illness, the degree of inflammation and vascular permeability can differ substantially between patients with acute respiratory failure, including those with acute respiratory distress syndrome (ARDS) [14–16].

The present study was conducted to evaluate the relationship of systemic inflammation and vascular permeability markers with respiratory drive among patients with ARDS. We hypothesized that greater inflammation and endothelial barrier dysfunction are associated with higher respiratory drive and may help explain drive heterogeneity in patients with ARDS after accounting for other clinically observable factors. We also evaluated the association of respiratory drive with clinical outcomes.

## Methods

### Study participants

Patients enrolled in the multicenter EPVent-2 trial (NCT01681225) were evaluated for inclusion in this sub-study. Eligibility criteria for the parent trial have been published previously [17] and include patients aged at least 16 years and undergoing invasive ventilation for early moderate or severe ARDS (PaO_2_/FiO_2_ ≤ 200 mm Hg). The trial compared two strategies for titrating positive end-expiratory pressure (PEEP): one guided by oxygenation and esophageal pressure combined, and one guided by oxygenation alone. There was no significant difference in primary or secondary clinical endpoints between treatment groups. All trial participants, regardless of randomized treatment allocation, underwent esophageal manometry per trial protocol.

Trial participants were excluded from this sub-study if esophageal pressure waveform recordings were unavailable for analysis at baseline or within one hour of enrollment, the period of interest for this analysis. Trial participants also were excluded if plasma biomarkers of inflammation were not obtained, or if the patient received neuromuscular blockade during the measurements of interest.

### Measure of respiratory drive

Airway flow, airway pressure, and esophageal pressure were measured as previously described [17] using dedicated equipment for signal acquisition. Waveforms were recorded at a sampling rate of 60 Hz. The esophageal manometry catheter was positioned with the balloon in the mid-thoracic retrocardiac esophagus; placement was confirmed by visualization of cardiac pressure oscillations and a confirmatory maneuver consisting of chest wall push with airway occlusion during which an approximately 1:1 change in airway:esophageal pressure was observed.

Respiratory drive was measured using P_ES_0.1, the change in esophageal pressure during the first 0.1 s (100 ms) of patient inspiratory effort [18]. Inspiratory effort was detected by the abrupt negative deflection in slope of the P_ES_-time tracing just prior to onset of a machine inspiratory cycle. Only efforts that resulted in triggering of a machine inspiratory cycle were considered.

P_ES_0.1 measurements were performed on three representative respiratory cycles and the average result reported. Representative cycles were characterized by the absence of recent coughing, swallowing, or esophageal spasm.

### Vascular permeability, inflammation, and respiratory drive

Plasma angiopoietin-2 (angpt-2) was measured as a vascular permeability marker [13, 19, 20]. Plasma cytokines interleukin-6 (IL-6) and interleukin-8 (IL-8) were measured as pro-inflammatory markers [13, 14]. Blood plasma samples were obtained at trial enrollment. Analytes were measured using the ProteinSimple Ella, a microfluidic multi-analyte immunoassay platform. Plasma biomarker concentrations were measured in triplicate, and the average value was used for analyses. Values were log-transformed for entry into models.

To visualize the crude relationship between plasma biomarkers and respiratory drive, the range of P_ES_0.1 across tertiles of biomarker values was examined. The association of plasma biomarkers with respiratory drive was further evaluated with a series of models. Respiratory drive, measured using P_ES_0.1, was expected to have a semi-continuous distribution with zero-inflation and right-skewed positive values. To account for this distribution, we used marginalized two-part (MTP) models, modeling first the probability of having a zero-value, and then modeling the non-zero outcomes on a continuous positive domain [21, 22], assuming a log-normal distribution. Covariates were entered for the non-zero continuous model, while an intercept-only model was used for the zero distribution.

With this statistical approach, a series of unadjusted models were developed entering each plasma biomarker as the independent variable. Then, a series of multivariable models were developed adjusting for variables known to modulate respiratory drive: mechanoreceptor inputs (end-inspiratory transpulmonary pressure, end-expiratory transpulmonary pressure, and tidal volume scaled to predicted body weight), chemoreceptor inputs (pH, PaCO_2_, PaO_2_), and suprapontine input (sedation depth measured with Richmond Agitation-Sedation Scale [RASS]) [1, 8, 23, 24]. Although pain/discomfort is a potential contributor to respiratory drive, no measure of pain/discomfort was recorded in the trial, and thus it was not included in analyses.

Sensitivity analyses were performed replacing transpulmonary pressure with airway driving pressure and PEEP, routinely clinically available measures. Similarly, additional models adjusted for clinically available surrogates of inflammation (temperature, white blood cell count) and organ injury (sequential organ failure assessment [SOFA]) to determine whether associations of plasma biomarkers with drive might be explained by clinically available data. To scrutinize robustness independent of modeling approach, analyses were repeated using hurdle models.

### Sedation-drive discordance

Marginalized two-part models as described above were also used to evaluate the relationship between sedation depth and respiratory drive. First, the unadjusted association of RASS with P_ES_0.1 was evaluated. Then, a multivariable model was developed adjusting for clinically observable factors known to modulate respiratory drive: mechanics, chemoreceptor inputs, and suprapontine input, as described above.

Evaluation for potential confounding by vascular permeability or inflammation—which are not readily quantified clinically—was then evaluated in two series of models. First, plasma biomarkers were added to the multivariable marginalized two-part models to establish their association with respiratory drive and determine whether the relationship of sedation depth with respiratory drive changed after adjusting for between-patient differences in vascular permeability or systemic inflammation. Second, linear regression was used to explore whether vascular permeability or inflammation also correlated with sedation depth.

### Respiratory drive and clinical outcomes

The relationship between respiratory drive and favorable clinical outcomes was hypothesized to be U-shaped, with the best outcome anticipated among patients with moderate drive compared to those with high or low drive. To accommodate this non-linear relationship, patients were classified as having low drive if P_ES_0.1 was less than 0.5 cm H_2_O, moderate drive if between 0.5 and 2.9 cm H_2_O, and high drive if 3.0 cm H_2_O or higher, values selected a priori based on available literature [8, 25].

The unadjusted association between respiratory drive class and 60-day mortality was visualized with Kaplan–Meier plots, which were compared using the log-rank test. Unadjusted Cox proportional hazards models were used to provide effect estimates for the association of drive class with 60-day mortality. Multivariable models were developed to account for potential confounders and well-established prognostic factors, incorporating measures of lung injury severity and multiorgan failure to improve effect estimate precision. The primary multivariable model adjusted for study arm and baseline values of airway driving pressure, PaO_2_/FiO_2_, ventilatory ratio (a surrogate of dead-space fraction [26]), and non-pulmonary sequential organ failure assessment (SOFA). Sensitivity analyses were conducted using more parsimonious and more expansive covariate adjustments (Additional file 1). The proportional hazards assumption was assessed per the method of Lin, Wei, and Ying [27], and by plotting Schoenfeld residuals versus time for the covariate of interest. For all Cox models, the Wald type-3 test was used to test the effect of P_ES_0.1 class as a 3-level non-ordinal class variable.

Ventilator-free days were computed per the ARDSNet method as the number of days between successful liberation from mechanical ventilation and study day 28, assigning a value of zero free days for non-survivors [28], The association between respiratory drive class and ventilator-free days was evaluated via zero-inflated Poisson regression. Covariate adjustment mirrored that for the survival analyses detailed above, and an intercept-only zero-model was assumed.

### Common statistical procedures

Analyses were performed using SAS 9.4. For all analyses, *p* < 0.05 was considered statistically significant without adjustment for multiple comparisons.

## Results

### Patient characteristics

Of 200 enrolled patients in the EPVent-2 trial, 2 patients were excluded for missing respiratory waveform recording files, 7 for missing plasma biomarkers, and 67 for neuromuscular blockade reported during waveform recordings. Thus, 124 patients enrolled across 12 sites were included in analyses (Additional file 1: Table E1). Their baseline characteristics are presented in Table 1 and Additional file 1: Table E2.

**Table 1:** Baseline characteristics of study participants

Variable	All patients (n = 124)	Respiratory drive present (n = 68)	Respiratory drive absent (n = 56)	Difference (95% CI)
Age, years	58 ± 15	61 ± 15	55 ± 16	5 (0–11)
Female	60 (48.4%)	35 (51.5%)	25 (44.6%)	6.8 (− 10.8 to 24.5)
Body mass index, kg/m^2^	33.1 ± 12.2	31.6 ± 12.5	35.0 ± 11.8	− 3.4 (− 7.8 to 0.9)
APACHE-II	27 ± 7	26 ± 7	28 ± 7	− 2 (− 5 to 0)
SOFA	11 ± 4	10 ± 3	12 ± 4	− 2 (− 3 to 0)
Duration of invasive ventilation prior to enrollment, hours	23 ± 12	21 ± 12	25 ± 13	-4 (-8 to 0)
Concomitant diagnoses				
Pneumonia	94 (75.8%)	50 (73.5%)	44 (78.6%)	− 5.0 (− 20.1 to 10.0)
Sepsis	109 (87.9%)	59 (86.8%)	50 (89.3%)	− 2.5 (− 13.9 to 8.9)
Shock requiring vasopressor or inotrope	68 (54.8%)	33 (48.5%)	35 (62.5%)	− 14.0 (− 31.4 to 3.4)
Sedation depth, Richmond agitation-sedation scale (RASS)	− 3 [− 4 to − 2]	− 3 [− 4 to − 2]	− 4 [− 4 to − 2]	0 (0–1)
Arterial blood gas				
pH	7.34 ± 0.08	7.36 ± 0.08	7.33 ± 0.09	0.03 (0.00 to 0.06)
PaCO_2_, mm Hg	44 ± 11	42 ± 9	45 ± 13	-2 (-6 to 2)
PaO_2_, mm Hg	78 ± 24	75 ± 18	81 ± 30	-5 (-14 to 3)
PaO_2_:FiO_2_	107 ± 37	106 ± 32	107 ± 42	− 1 (− 14 to 12)
Tidal volume, mL	398 ± 73	384 ± 67	415 ± 77	− 31 (− 57 to − 5)
Tidal volume, mL/kg PBW	6.5 ± 1.1	6.5 ± 1.0	6.6 ± 1.1	− 0.1 (− 0.5 to 0.3)
Set PEEP, cm H_2_O	13 ± 4	12 ± 4	14 ± 4	− 2 (− 3 to 0)
Respiratory rate, breaths/min	25 ± 5	25 ± 5	25 ± 5	0 (− 1 to 2)
Minute ventilation, L/min	9.9 ± 2.5	9.8 ± 2.3	10.1 ± 2.7	− 0.3 (− 1.2 to 0.5)
Ventilatory ratio	1.9 ± 0.5	1.9 ± 0.5	1.9 ± 0.5	0.0 (− 0.2 to 0.2)
Mechanics				
Plateau pressure, cm H_2_O	27 ± 5	26 ± 6	28 ± 5	− 2 (− 4 to − 1)
Airway driving pressure, cm H_2_O	13 ± 4	13 ± 4	13 ± 3	0 (− 2 to 1)
Lung end-inspiratory pressure, cm H_2_O	8 ± 5	8 ± 5	7 ± 5	2 (0–4)
Lung end-expiratory pressure, cm H_2_O	− 2 ± 4	− 1 ± 4	− 2 ± 5	1 (0–3)
Respiratory system compliance, cm H_2_O	34 ± 14	34 ± 17	33 ± 10	1 (− 4 to 6)
Lung compliance, mL/cm H_2_O	50 ± 25	49 ± 25	52 ± 25	− 2 (− 11 to 7)
Assigned to esophageal pressure-guided PEEP trial arm	62 (50.0%)	31 (45.6%)	31 (55.4%)	− 9.8 (− 27.4 to 7.8)

Sixty-eight of the 124 included patients (54.8%) had respiratory drive detectable with P_ES_0.1 during the first hour of enrollment. Among those with detectable drive, the mean P_ES_0.1 was 2.4 (standard deviation 1.2, maximum 5.1) cm H_2_O.

### Inflammation, vascular permeability, and respiratory drive

**Fig. 1:**
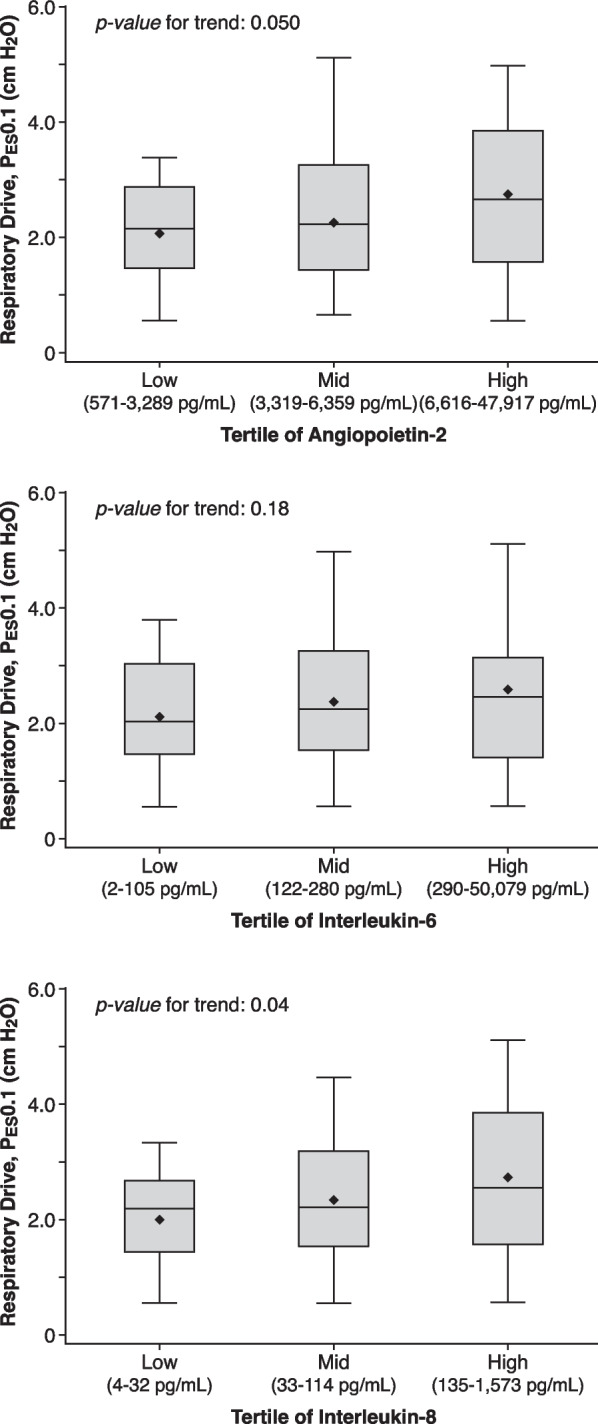
Association of circulating inflammatory biomarkers angiopoietin-2, interleukin-6, and interleukin-8, with respiratory drive. To facilitate data visualization, the range of P_ES_0.1 across tertiles of biomarker values is shown

**Fig. 2:**
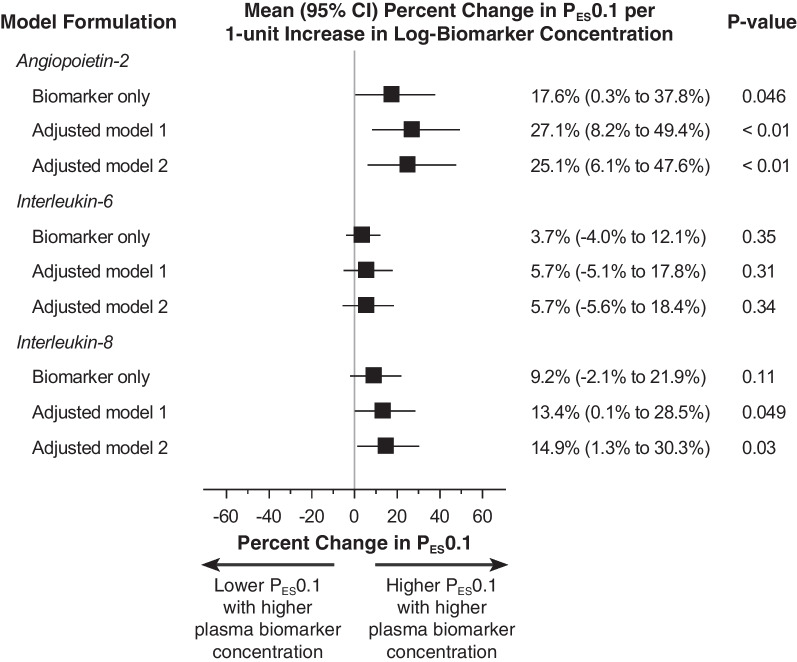
Association of circulating plasma concentration of inflammatory biomarkers angiopoietin-2, interleukin-6, and interleukin-8, with respiratory drive in univariable and multivariable models. Adjusted model 1 includes the biomarker of interest and measures of lung mechanics (tidal volume scaled to predicted body weight, end-inspiratory transpulmonary pressure, end-expiratory transpulmonary pressure), chemoreceptor input (pH, PaCO_2_, PaO_2_) and sedation depth (Richmond agitation-sedation score [RASS]). Adjusted model 2 includes all covariates in adjusted model 1 plus clinical markers of inflammation (maximum temperature and white blood cell count in the 24 h preceding enrollment) and an index of multiorgan dysfunction (sequential organ failure assessment [SOFA]). The biomarker of interest was entered as a continuous log-transformed variable into each model. Additional sensitivity analyses with alternative model formulations are reported in the online supplement

The association of plasma biomarkers with P_ES_0.1 is shown in Figs. 1 and 2.

Angiopoietin-2 entered as a continuous variable was significantly associated with respiratory drive (17.6% increase in P_ES_0.1 per 1-unit increase in log-angiopoietin-2) in unadjusted analysis. Higher angiopoietin-2 remained significantly associated with higher drive in a multivariable model adjusting for mechanics, chemoreceptor input, and sedation depth.

IL-6 was not significantly associated with respiratory drive in unadjusted or adjusted models.

IL-8 was not significantly associated with respiratory drive in an unadjusted model. However, after adjusting for mechanics, chemoreceptor input, and sedation depth, higher IL-8 was also significantly associated with higher respiratory drive.

Sensitivity analyses demonstrated robustness of findings for angiopoetin-2, IL-6, and IL-8 to alternative modeling approaches and entering alternative measures of respiratory mechanics and clinical surrogates of inflammation that are routinely monitored in clinical practice (Additional file 1: Tables E3 and E4).

### Sedation depth and respiratory drive

Sedation depth assessed via RASS ranged between unarousable (RASS −5) to awake and agitated (RASS +2), with a median RASS of −3 (interquartile range −4 to −2). Sedation depth was not significantly associated with P_ES_0.1 in an unadjusted model, or in a multivariable model adjusting for mechanics and chemoreceptor input (Table 2). However, after plasma biomarkers angiopoietin-2, IL-6, or IL-8 were added to models, lighter sedation (higher RASS) was significantly associated with higher P_ES_0.1 (Table 2). Similar findings were observed in sensitivity analyses replacing measures of transpulmonary pressure with routinely clinically available measures of mechanics: the positive correlation of RASS with drive was statistically significant only after adjusting for plasma biomarkers of vascular permeability or inflammation (Additional file 1: Table E5).

**Table 2:** Association of RASS with respiratory drive in marginalized two-part models

Model specification	Model AIC^a^	Percent change in P_ES_0.1 per 1-unit change in RASS	95% CI for RASS	*p* value for RASS
RASS only	336.3	5.9%	− 2.9 to 15.5%	0.19
Core multivariable model adjusting for mechanics, chemoreceptor input, and sedation depth^a^	318.9	9.2%	− 0.5 to 19.7%	0.06
Core multivariable model^b^ plus angiopoetin-2^c^	312.4	11.7%	2.5–21.7%	0.01
Core multivariable model^b^ plus interleukin-6^c^	320.2	11.4%	0.7% to 23.4%	0.04
Core multivariable model^b^ plus interleukin-8^c^	317.4	12.9%	2.6–24.3%	0.01

^a^AIC refers to Akaike information criterion, an index of how well the model fits the data. AIC is calculated from the model’s maximum likelihood and includes a penalty for increasing the number of independent variables in the model. Lower AIC signifies better model fit

^b^Core multivariable model includes the following covariates: tidal volume per predicted body weight, end-inspiratory transpulmonary pressure, end-expiratory transpulmonary pressure, pH, PaCO_2_, PaO_2_, Richmond agitation-sedation score (RASS)

^c^Coefficients for biomarkers in this model are presented in Table 2. Angiopoietin-2 and IL-8 were also significantly associated with P_ES_0.1

To evaluate the role of vascular permeability and inflammation as potential confounders between sedation depth and respiratory drive, the unadjusted correlation between plasma biomarkers and RASS also was evaluated. RASS was significantly inversely correlated with IL-6 (Pearson* r*: −0.36, 95% CI − 0.52 to − 0.17; *p* < 0.01) and IL-8 (Pearson* r*: −0.29, 95% CI − 0.47 to − 0.10; *p* < 0.01), but not angiopoietin-2 (Pearson* r*: −0.14, 95% CI − 0.33 to 0.07; *p* = 0.18), indicating deeper sedation depth was observed among patients with a more pro-inflammatory immune response.

### Respiratory drive and clinical outcomes

Low respiratory drive, defined as P_ES_0.1 < 0.5 cm H_2_O, was observed in 45% of patients, all of whom had P_ES_0.1 of 0 cm H_2_O indicative of no drive. Moderate respiratory drive, defined as P_ES_0.1 of 0.5–2.9 cm H_2_O, was observed in 38% of patients, in whom median P_ES_0.1 was 1.6 (interquartile range 1.3–2.3) cm H_2_O. High respiratory drive, defined as P_ES_0.1 ≥ 3.0 cm H_2_O, was observed in 17% of patients, in whom median P_ES_0.1 was 3.4 (interquartile range 3.2–4.4) cm H_2_O.

**Fig. 3:**
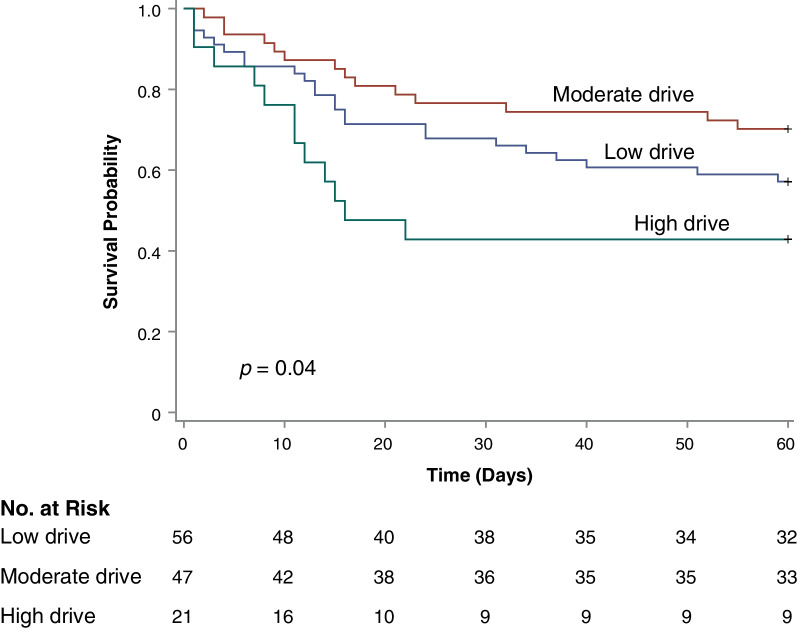
Respiratory drive and survival. Kaplan–Meier plots. Patients were classified as having low drive if P_ES_0.1 was less than 0.5 cm H_2_O, moderate drive if between 0.5–2.9 cm H_2_O, and high drive if 3.0 cm H_2_O or higher. P_ES_0.1 refers to the change in esophageal pressure, a surrogate of pleural pressure, during the first 0.1 s (100 ms) of patient inspiratory effort

Mortality differed significantly by level of respiratory drive (Kaplan–Meier log-rank *p* = 0.04) and was lowest among patients with moderate respiratory drive (Fig. 3). Compared to patients with moderate drive (P_ES_0.1 of 0.5–2.9 cm H_2_O), the hazard ratio for death for patients with lower drive was 1.58, 95% CI 0.82–3.05, while the hazard ratio for death for patients with higher drive was 2.63, 95% CI 1.21–5.70 (*p* = 0.049 for 3-level non-ordinal class variable).

In the main multivariable model, level of respiratory drive remained significantly associated with 60-day mortality after adjusting for treatment group, airway driving pressure, PaO_2_/FiO_2_, ventilatory ratio, and non-pulmonary SOFA. Compared to patients with moderate drive, the hazard ratio for death with lower drive was 1.36, 95% CI 0.68–2.72, while the hazard ratio for death with higher drive was 2.62, 95% CI 1.20–5.73 (*p* = 0.049 for 3-level non-ordinal class variable). The relationship between level of respiratory drive and mortality remained statistically significant and qualitatively similar in sensitivity analyses with more parsimonious and more extensive covariate formulations, including after adjustment for biomarker plasma levels (Additional file 1: Table E6). Mortality was lowest among patients with moderate respiratory drive consistently across analyses.

Ventilator-free days did not differ significantly by level of respiratory drive in an unadjusted zero-inflated Poisson model (*p* = 0.09). However, in the main multivariable model, level of respiratory drive was significantly associated with ventilator-free days after adjusting for treatment group, airway driving pressure, PaO_2_/FiO_2_, ventilatory ratio, and non-pulmonary SOFA. In this model, ventilator-free days appeared highest among patients with the highest respiratory drive class. The difference between low versus moderate drive appeared negligible. A similar pattern was observed across sensitivity analyses (Additional file 1: Table E7).

## Discussion

To our knowledge, this study is the first to investigate the association between vascular permeability, systemic inflammation, and respiratory drive in patients with acute respiratory failure. Three major findings are of note. First, greater vascular permeability and systemic inflammation, signified by circulating angiopoietin-2 and IL-8, were associated with higher respiratory drive independent of known drive-determining factors. In unadjusted analysis, however, only angiopoietin-2, a marker of vascular permeability, was correlated with respiratory drive. Second, heterogeneity in respiratory drive at a given sedation depth was explained in part by differences in vascular permeability and systemic inflammation. Finally, patients with moderate respiratory drive had lower 60-day mortality than patients with either low or high drive.

At least three overarching mechanisms exist by which vascular permeability and systemic inflammation may influence ventilatory control.

First, some cytokines may directly access the brain: by active transport and/or traversing the modified barrier of the circumventricular organs [29–33]. Blood–brain barrier dysfunction is common in critical illness and may be mediated in part by angiopoietin-2 [34–36], a potent regulator of endothelial barrier function and vascular permeability that is often elevated in critical illness [19, 20, 37].

Second, inflammatory signals may be transmitted neurally across the blood–brain barrier by peripheral neurosensory afferents of the vagus and carotid sinus nerves, which express cytokine receptors at the viscera and carotid body, respectively [38–41]. In the present study, higher plasma levels of angiopoietin-2 and IL-8 were correlated with higher drive, of potential relevance to both mechanisms. Robust inflammation and vascular permeability may also indirectly influence drive through effects on gas exchange, perfusion, and respiratory mechanics.

Third, vascular permeability and systemic inflammation may be part of a positive feedback loop as higher respiratory drive contributes to patient self-inflicted lung injury (P-SILI), for example through high tidal volumes, breath stacking, and regional strain/pendelluft [4, 42–44]. P-SILI in turn causes additional inflammation and vascular injury, increasing respiratory drive further. This resultant positive feedback loop is potentially deleterious.

**Fig. 4:**
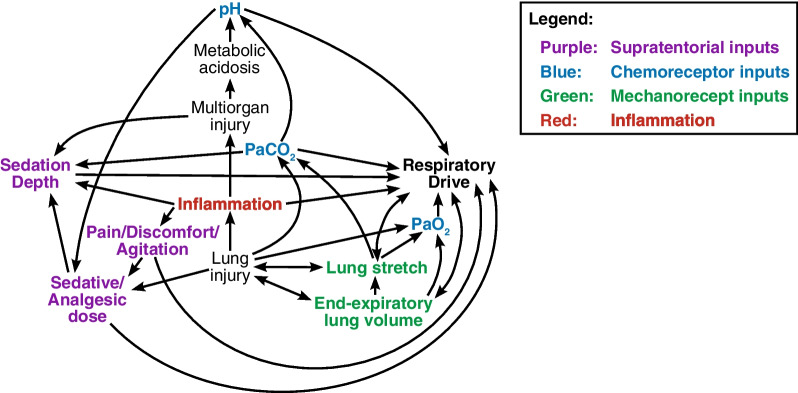
Factors contributing to respiratory drive heterogeneity in acute respiratory failure. Inflammation and associated vascular permeability are key determinants of respiratory drive in critical illness that may explain why some critically ill patients experience refractory high drive despite accounting for other clinically observable factors, including even deep sedation. High drive in turn may cause patient self-inflicted lung injury (P-SILI) that increases inflammatory signaling, creating a positive feedback loop. Blue denotes chemoreceptor inputs. Green denotes pulmonary mechanoreceptor inputs. Purple denotes supratentorial inputs. Red denotes inflammatory input

The potential role of systemic inflammation and vascular permeability as occult determinants of respiratory drive also bears clinical relevance in light of the routine practice [45–47], often ineffective [1, 48], of deepening sedation in attempt to achieve respirolysis. Respiratory drive can differ considerably between critically ill patients at any given sedation depth [1], and such heterogeneity is not fully explained by routinely clinically measured factors (e.g. pH, PaCO_2_, PaO_2_, respiratory mechanics, sedation depth, pain). This study identifies systemic inflammation and vascular permeability as potential sources of drive heterogeneity. Therefore, sedatives and analgesics that are potent respirolytics in healthy individuals [49–53] may not consistently attenuate drive in critical illness in part because hyperinflammatory states and endothelial barrier dysfunction can differ between patients [14, 15, 54]. A theoretical depiction of the complex relationships contributing to respiratory drive in critical illness is shown in Fig. 4.

This study observed that ARDS patients with moderate drive had lower mortality than those with lower or higher drive. Mechanistically, low drive could predispose to diaphragm disuse atrophy and atelectasis (from non-variable tidal insufflation), and signify depressed cough reflex that impedes secretion clearance [2, 3]. High drive may cause injury through greater lung strain and diaphragm load [55]. However, risks of lung and diaphragm injury undoubtedly are nonuniform between patients, even in ARDS. Spontaneous breathing of course is a prerequisite for liberation from invasive mechanical ventilation and may explain the association between higher baseline drive and more ventilator-free days in this observational cohort—though notably high drive also was associated with higher mortality.

### Limitations

Noteworthy limitations of this study include the following. This analysis relied on observational data for which causality cannot be ascertained. The relationship of respiratory drive with inflammation, endothelial injury, and other drive determinants is undoubtedly complex and, in some cases, may be bidirectional. For example, high respiratory drive may increase inflammation and endothelial permeability through P-SILI (potential reverse causation). We would speculate an injurious positive feedback loop between inflammation/endothelial permeability, respiratory drive, and lung injury is most likely. This study’s cross-sectional design also precludes evaluation of the *within-patient* relationship between changes in systemic inflammation, endothelial permeability, and respiratory drive.

No index of pain was recorded in the dataset. While thought to be infrequent with current best practice, untreated pain could contribute to increased respiratory drive.

The clinical trial from which data were obtained did not prospectively measure respiratory drive, which was instead quantified post-hoc via P_ES_0.1 from waveform recordings. Other techniques for quantifying drive, such as P_airway_0.1, pressure–time product, or work of breathing, were not available because they require specific ventilator maneuvers and/or observation of passive chest wall mechanics.

This hypothesis-generating analysis did not exhaustively interrogate inflammatory or vascular pathways. Other pro-inflammatory, anti-inflammatory and endothelial/vascular biomarkers, including cytokines, reactive oxygen species, and signaling pathways that may be pertinent to respiratory drive were not measured. Which circulating molecules and pathways are mechanistically linked—potentially relevant for targeted intervention—remains to be determined.

This study’s definition for moderate drive (P_ES_0.1 0.5–2.9 cm H_2_O) was formulated to account for the upper limit beyond which accessory inspiratory muscle use may occur [8, 25], thus approximating the normal range in health. Mechanisms by which extremes of respiratory drive might worsen clinical outcomes were not evaluated in this study, and it remains possible this association is largely an epiphenomenon signifying illness severity.

Unpacking the determinants and clinical significance of respiratory drive is a complicated exercise given the complex pathways modulating control of breathing, inflammation, and endothelial barrier function, and the dynamic nature of both respiratory drive and critical illness. This study highlights inflammation and vascular permeability as potential contributors to respiratory drive heterogeneity and redemonstrates an association between respiratory drive extremes and untoward clinical outcomes. However, it does not provide sufficient insight to guide clinical care. Whether targeting any particular range of respiratory drive is beneficial in acute respiratory failure remains untested.

## Conclusions

In this cross-sectional study of critically ill adults with ARDS, markers of systemic inflammation and vascular permeability were independently associated with higher respiratory drive. These data suggest the heterogeneous response of respiratory drive to sedation may be explained in part by heterogeneity in systemic inflammation and vascular endothelial barrier dysfunction during critical illness. Patients with moderate respiratory drive, approximating the normal physiologic range, experienced lowest risk of death. Whether therapeutically targeting any particular level of respiratory drive affords clinical benefit remains untested.

### Supplementary Information

**Additional file 1.** Supplementary E-tables.

## Data Availability

The datasets used during the current study are available from the corresponding author.
