# Supplementary material for: Respiratory drive heterogeneity associated with systemic inflammation and vascular permeability in acute respiratory distress syndrome
Source: Crit Care. 2024 Apr 23;28:136. doi: 10.1186/s13054-024-04920-4 (PMC11036740; doi:10.1186/s13054-024-04920-4)
Supplement: Supplementary file 1 — Additional file 1. Supplementary E-tables. [file 13054_2024_4920_MOESM1_ESM.docx]

**Online Supplement**

**Respiratory Drive Heterogeneity Associated with Systemic Inflammation and Vascular Permeability in Acute Respiratory Distress Syndrome**

Elias Baedorf-Kassis, MD

Michael Murn, MD

Amy Dzierba, PharmD

Alexis Serra, MD, MPH

Ivan Garcia, RRT

Emily Minus, BA

Clarissa Padilla, MPH

Todd Sarge, MD

Valerie M. Goodspeed, MPH

Michael A. Matthay, MD

Michelle N. Gong, MD, MS

Deborah Cook, MD

Stephen H. Loring, MD

Daniel Talmor, MD, MPH

Jeremy R. Beitler, MD, MPH

for the EPVent-2 Study Group

**Table of Contents**

| **Content** | **Pages** |
| --- | --- |
| EPVent-2 Study Group investigators | 3 |
| Table E1. Characteristics of EPVent2 Trial Participants Included vs. Excluded from this Study | 4 |
| Table E2. Baseline Characteristics of Included Participants by Respiratory Drive Class | 5 |
| Table E3. Sensitivity Analyses for Association of Inflammatory Biomarkers with Respiratory Drive (P_ES_0.1) Adjusting for Routinely Clinically Available Measures: Marginalized Two-Part Models | 6 |
| Table E4. Sensitivity Analyses for Association of Inflammatory Biomarkers with Respiratory Drive (P_ES_0.1): Hurdle Models | 7 |
| Table E5. Association of RASS with Respiratory Drive (PES0.1) Adjusting for Routinely Clinically Available Measures: Marginalized Two-Part Models | 8 |
| Table E6. Association of Respiratory Drive (P_ES_0.1) with 60-Day Mortality, Presenting by Increasing Model Complexity | 9 |
| Table E7. Association of Respiratory Drive (P_ES_0.1) with Ventilator-Free Days: Zero-Inflated Poisson Models | 10 |

**List of EPVent-2 Site Investigators**

| **Site** | **Location** | **Principal Investigator** | **Study Coordinator & Respiratory Therapist** |
| --- | --- | --- | --- |
| Beth Israel Deaconess Medical Center, Harvard Medical School | Boston, MA, USA | Daniel Talmor, MD, MPH  Todd Sarge, MD | Valerie Banner-Goodspeed, MPH  Emily Fish, MD, MPH  Sayuri Jinadasa, MD, MPH  Ray Ritz, RRT, FAARC  Joseph Previtera, RRT |
| Montefiore Medical Center, Albert Einstein College of Medicine | Bronx, NY, USA | Michelle N. Gong, MD, MSc | Lawrence Lee, PhD, PA-C |
| University of California San Diego | San Diego, CA, USA | Jeremy R. Beitler, MD, MPH |  |
| St. Joseph’s Healthcare, McMaster University | Hamilton, ON, Canada | Deborah Cook, MD, MS | France Clarke, RRT  Tom Piraino, RRT |
| Stanford University | Palo Alto, CA, USA | Joseph Levitt, MD, MS | Rosemary Vojnik, BS |
| University of Michigan | Ann Arbor, MI, USA | Pauline Park, MD, FACS, FCCM | Kristin Brierley, CCRP  Carl Haas MLS, RRT-ACCS, FAARC  Andrew Weirauch BS, RRT-ACCS |
| Toronto General Hospital, University of Toronto | Toronto, ON, Canada | Eddy Fan, MD, FRCPC, PhD | Andrea Matte, RRT |
| Massachusetts General Hospital, Harvard Medical School | Boston, MA, USA | R. Scott Harris, MD | Mamary Kone, MD, MPH |
| University of Massachusetts | Worcester, MA, USA | Stephen Heard, MD | Karen Longtine, BS, RN, CCRC |
| Université Laval | Quebec City, QC, Canada | François Lellouche, MD, PhD | Pierre-Alexandre Bouchard, RRT |
| R. Adams Cowley Shock Trauma Center, University of Maryland | Baltimore, MD, USA | Lewis Rubinson, MD, PhD, FCCP | Jennifer (Titus) McGrain, RRT |
| Vancouver General Hospital | Vancouver, BC, Canada | Donald E G Griesdale, MD, MPH, FRCPC | Denise Foster, RN, CCRP |
| Mayo Clinic | Rochester, MN, USA | Richard Oeckler, MD, PhD | Amy Amsbaugh RRT, RCP |
| Orlando Health, Inc. | Orlando, FL, USA | Edgar Jimenez, MD, FCCM | Valerie Danesh, RN, BSN, MHSA, CCRP |

| **Table E1. Characteristics of EPVent2 Trial Participants Included vs. Excluded from this Study** | | |
| --- | --- | --- |
|  |  |  |
| **Variable** | **Included**  **(n = 124)** | **Excluded**  **(n = 76)** |
| Age, years | 58 ± 15 | 51 ± 16 |
| Female | 60 (48.4%) | 31 (41.9%) |
| Body mass index, kg/m^2^ | 33.1 ± 12.2 | 32.9 ± 11.5 |
| APACHE-II | 27 ± 7 | 28 ± 8 |
| SOFA | 11 ± 4 | 11 ± 4 |
| Duration of invasive ventilation prior to enrollment, hours | 23 ± 12 | 26 ± 19 |
| Concomitant diagnoses |  |  |
| Pneumonia | 94 (75.8%) | 53 (71.6%) |
| Sepsis | 109 (87.9%) | 60 (81.1%) |
| Shock requiring vasopressor or inotrope | 68 (54.8%) | 45 (60.8%) |
| Arterial blood gas |  |  |
| pH | 7.34 ± 0.08 | 7.28 ± 0.09 |
| PaCO_2_, mm Hg | 44 ± 11 | 47 ± 12 |
| PaO_2_, mm Hg | 78 ± 24 | 70 ± 16 |
| PaO_2_:FiO_2_ | 107 ± 37 | 87 ± 32 |
| Tidal volume, mL | 398 ± 73 | 389 ± 97 |
| Tidal volume, mL/kg PBW | 6.5 ± 1.1 | 6.2 ± 1.1 |
| Set PEEP, cm H_2_O | 13 ± 4 | 15 ± 3 |
| Respiratory rate, breaths/min | 25 ± 5 | 28 ± 6 |
| Minute ventilation, L/min | 9.9 ± 2.5 | 10.8 ± 3.2 |
| Ventilatory ratio | 1.9 ± 0.5 | 2.2 ± 0.8 |
| Mechanics |  |  |
| Plateau pressure, cm H_2_O | 27 ± 5 | 30 ± 5 |
| Airway driving pressure, cm H_2_O | 13 ± 4 | 13 ± 4 |
| Lung end-inspiratory pressure, cm H_2_O | 8 ± 5 | 10 ± 4 |
| Lung end-expiratory pressure, cm H_2_O | -2 ± 4 | 0 ± 4 |
| Respiratory system compliance, cm H_2_O | 34 ± 14 | 33 ± 14 |
| Lung compliance, mL/cm H_2_O | 50 ± 25 | 46 ± 17 |
| Assigned to esophageal pressure-guided PEEP trial arm | 62 (50.0%) | 39 (52.7%) |
| Plasma biomarkers |  |  |
| Angiopoietin-2, pg/mL | 5206 [2918-8902] | 7283 [4361-13,974] |
| Interleukin-6, pg/mL | 166 [66-615] | 435 [103-2027] |
| Interleukin-8, pg/mL | 51 [25-153] | 71 [36-202] |
| Mortality through day 60 | 51 (40.8%)^a^ | 24 (32.4%) |
| Ventilator-free days through day 28 | 17.5 [0-23] | 16.5 [0-22] |
| ^a^ One trial participant was lost to follow-up between day 28 and day 60. | | |

| **Table E2. Baseline Characteristics of Included Participants by Respiratory Drive Class** | | | |
| --- | --- | --- | --- |
| **Variable** | **Low Respiratory drive**  **(n = 56)** | **Moderate Respiratory Drive**  **(n = 47)** | **High Respiratory Drive**  **(n = 21)** |
| Age, years | 55 ± 16 | 59 ± 15 | 64 ± 14 |
| Female | 25 (44.6%) | 24 (51.1%) | 11 (52.4%) |
| Body mass index, kg/m^2^ | 35.0 ± 11.8 | 33.3 ± 14.0 | 27.7 ± 6.7 |
| APACHE-II | 28 ± 7 | 25 ± 7 | 28 ± 8 |
| SOFA | 12 ± 4 | 10 ± 3 | 11 ± 3 |
| Duration of invasive ventilation prior to enrollment, hours | 25 ± 13 | 18 ± 9 | 27 ± 15 |
| Concomitant diagnoses |  |  |  |
| Pneumonia | 44 (78.6%) | 35 (74.5%) | 15 (71.4%) |
| Sepsis | 50 (89.3%) | 41 (87.2%) | 18 (85.7%) |
| Shock requiring vasopressor or inotrope | 35 (62.5%) | 25 (53.2%) | 8 (38.1%) |
| Sedation depth, Richmond agitation-sedation scale (RASS) | -4 [-4 to -2] | -3 [-4 to -2] | -3 [-4 to -2] |
| Arterial blood gas |  |  |  |
| pH | 7.33 ± 0.09 | 7.35 ± 0.07 | 7.37 ± 0.08 |
| PaCO_2_, mm Hg | 45 ± 13 | 43 ± 9 | 41 ± 9 |
| PaO_2_, mm Hg | 81 ± 30 | 76 ± 20 | 74 ± 10 |
| PaO_2_:FiO_2_ | 107 ± 42 | 103 ± 33 | 113 ± 32 |
| Tidal volume, mL | 415 ± 77 | 383 ± 68 | 385 ± 66 |
| Tidal volume, mL/kg PBW | 6.6 ± 1.1 | 6.5 ± 1.1 | 6.5 ± 0.9 |
| Set PEEP, cm H_2_O | 14 ± 4 | 13 ± 4 | 11 ± 4 |
| Respiratory rate, breaths/min | 25 ± 5 | 25 ± 5 | 26 ± 5 |
| Minute ventilation, L/min | 10.1 ± 2.7 | 9.5 ± 2.1 | 10.3 ± 2.7 |
| Ventilatory ratio | 1.9 ± 0.5 | 1.9 ± 0.5 | 1.9 ± 0.5 |
| Mechanics |  |  |  |
| Plateau pressure, cm H_2_O | 28 ± 5 | 26 ± 6 | 25 ± 6 |
| Airway driving pressure, cm H_2_O | 13 ± 3 | 13 ± 4 | 13 ± 4 |
| Lung end-inspiratory pressure, cm H_2_O | 7 ± 5 | 9 ± 5 | 8 ± 5 |
| Lung end-expiratory pressure, cm H_2_O | -2 ± 5 | -1 ± 4 | -1 ± 4 |
| Respiratory system compliance, cm H_2_O | 33 ± 10 | 35 ± 17 | 33 ± 17 |
| Lung compliance, mL/cm H_2_O | 52 ± 25 | 50 ± 27 | 48 ± 19 |
| Assigned to esophageal pressure-guided PEEP trial arm | 31 (55.4%) | 22 (46.8%) | 9 (42.9%) |

| **Table E3. Sensitivity Analyses for Association of Inflammatory Biomarkers with Respiratory Drive (P_ES_0.1) Adjusting for Routinely Clinically Available Measures: Marginalized Two-Part Models** | | | | |
| --- | --- | --- | --- | --- |
| **Model Specification and Biomarker of Interest** | **Model AIC** | **Percent Change in P_ES_0.1 per 1-unit change in Log-Biomarker**^a^ | **95% CI** | **p** |
| Clinical model: V_T_/PBW + ∆P + PEEP + pH + PaCO_2_ + PaO_2_ + RASS | 338.8 | -- | -- | -- |
| Clinical model + Angiopoetin-2^b^ | 327.9 | 34.7% | 15.4% to 57.1% | < 0.01 |
| Clinical model + Interleukin-6^c^ | 339.4 | 5.9% | -5.3% to 18.5% | 0.31 |
| Clinical model + Interleukin-8^d^ | 335.5 | 15.5% | 1.9% to 30.9% | 0.02 |
| ^a^ For reference, log-transformed values of biomarkers have the following mean ± SD (range): angpt2: 8.58 ± 0.89 (6.35-10.78); IL6: 5.23 ± 2.07 (0.72-11.95); IL8: 4.27 ± 1.53 (1.47-11.21); 8.48 ± 0.76 (6.81-10.68).  ^b^ Among other model covariates, lower ∆P, lower PaO_2_, and higher RASS (lighter sedation depth) were significantly correlated with higher respiratory drive in this multivariable model.  ^c^ Among other model covariates, higher RASS (lighter sedation depth) was significantly correlated with higher respiratory drive in this multivariable model.  ^d^ Among other model covariates, lower PaO_2_ and higher RASS (lighter sedation depth) were significantly correlated with higher respiratory drive in this multivariable model.  *Abbreviations*: V_T_/PBW, tidal volume in mL/kg predicted body weight; ∆P, airway driving pressure; PEEP, positive end-expiratory pressure; RASS, Richmond agitation-sedation scale. | | | | |

| **Table E4. Sensitivity Analyses for Association of Inflammatory Biomarkers with Respiratory Drive (P_ES_0.1): Hurdle Models** | | | | |
| --- | --- | --- | --- | --- |
| **Model Specification and Biomarker of Interest** | **Model AIC** | **Percent Change in P_ES_0.1 per 1-unit change in Log-Biomarker**^a^ | **95% CI** | **p** |
| Model A: No covariates | 392.2 | -- | -- | -- |
| Model A + Angiopoetin-2 | 390.3 | 17.6% | 0.5% to 37.6% | 0.04 |
| Model A + Interleukin-6 | 393.4 | 3.7% | -3.9% to 12.0% | 0.35 |
| Model A + Interleukin-8 | 391.7 | 9.2% | -2.0% to 21.7% | 0.11 |
| Model B: V_T_/PBW + P_L_,ins + P_L_,exp + pH + PaCO_2_ + PaO_2_ + RASS | 318.9 | -- | -- | -- |
| Model B + Angiopoetin-2^b^ | 312.2 | 28.5% | 9.6% to 50.7% | < 0.01 |
| Model B + Interleukin-6^c^ | 320.1 | 5.1% | -5.6% to 16.9% | 0.36 |
| Model B + Interleukin-8^b^ | 317.3 | 13.0% | -0.2% to 27.9% | 0.053 |
| Model C: V_T_/PBW + P_L_,ins + P_L_,exp + pH + PaCO_2_ + PaO_2_ + RASS + WBC + Tmax + SOFA | 322.6 | -- | -- | -- |
| Model C + Angiopoetin-2 | 317.4 | 26.1% | 7.1% to 48.4% | < 0.01 |
| Model C + Interleukin-6 | 323.9 | 4.7% | -6.4% to 17.1% | 0.42 |
| Model C + Interleukin-8 | 320.3 | 14.3% | 1.0% to 29.5% | 0.03 |
| Model D: V_T_/PBW + ∆P + PEEP + pH + PaCO_2_ + PaO_2_ + RASS | 338.8 | -- | -- | -- |
| Model D + Angiopoetin-2^d^ | 327.9 | 34.7% | 15.6% to 56.9% | < 0.01 |
| Model D + Interleukin-6^c^ | 339.2 | 7.4% | -3.8% to 20.0% | 0.20 |
| Model D + Interleukin-8^b^ | 335.4 | 16.3% | 2.8% to 31.6% | 0.02 |
| ^a^ For reference, log-transformed values of biomarkers have the following mean ± SD (range): angpt2: 8.58 ± 0.89 (6.35-10.78); IL6: 5.23 ± 2.07 (0.72-11.95); IL8: 4.27 ± 1.53 (1.47-11.21); 8.48 ± 0.76 (6.81-10.68).  ^b^ Among other model covariates, lower PaO_2_ and higher RASS (lighter sedation depth) also were significantly correlated with higher respiratory drive in this multivariable model.  ^c^ Among other model covariates, higher RASS (lighter sedation depth) was significantly correlated with higher respiratory drive in this multivariable model.  ^d^ Among other model covariates, lower ∆P, lower PaO_2_, and higher RASS (lighter sedation depth) also were significantly correlated with higher respiratory drive in this multivariable model.  *Abbreviations*: V_T_/PBW, tidal volume in mL/kg predicted body weight; P_L_,ins, end-inspiratory transpulmonary pressure; P_L_,exp, end-expiratory transpulmonary pressure; ∆P, airway driving pressure; PEEP, positive end-expiratory pressure; RASS, Richmond agitation-sedation scale; SOFA, sequential organ failure assessment; Tmax, maximum temperature in preceding 24 hours; WBC, maximum white blood cell count in preceding 24 hours. | | | | |

| **Table E5. Association of RASS with Respiratory Drive (P_ES_0.1) Adjusting for Routinely Clinically Available Measures: Marginalized Two-Part Models^a^** | | | | |
| --- | --- | --- | --- | --- |
| **Model Specification** | **Model AIC** | **Percent Change in P_ES_0.1 per 1-unit change in RASS** | **95% CI** | **p-value for RASS** |
| Clinical model^b^ | 338.8 | 9.0% | -1.6% to 20.6% | 0.10 |
| Clinical model^b^ plus angiopoetin-2 | 327.9 | 12.6% | 2.7% to 23.5% | 0.01 |
| Clinical model^b^ plus interleukin-6 | 339.4 | 11.9% | 0.4% to 24.8% | 0.04 |
| Clinical model^b^ plus interleukin-8 | 335.5 | 14.0% | 2.8% to 26.3% | 0.01 |
| ^a^ Coefficients for biomarkers in this model are presented in Figure 3 and Table E2. Angiopoietin-2 and Interleukin-8 were also significantly associated with P_ES_0.1.  ^b^ Clinical model includes the following covariates: tidal volume per predicted body weight, airway driving pressure, PEEP, pH, PaCO_2_, PaO_2_, Richmond agitation-sedation score. | | | | |

| **Table E6. Association of Respiratory Drive (P_ES_0.1) with 60-Day Mortality, Presenting by Increasing Model Complexity** | | |
| --- | --- | --- |
| **Model Specification** | **Hazard Ratio (95% CI)** | **p-value** |
| Respiratory drive class only |  |  |
| Low drive (ref: moderate drive) | 1.58 (0.82-3.05) | 0.049 |
| High drive (ref: moderate drive) | 2.63 (1.21-5.70) |  |
| Respiratory drive class + Study Arm + SOFA + ∆P |  |  |
| Low drive (ref: moderate drive) | 1.38 (0.69-2.75) | 0.03 |
| High drive (ref: moderate drive) | 2.76 (1.27-6.01) |  |
| Respiratory drive class + Study Arm + Non-pulmonary SOFA + PaO_2_/FiO_2_ + VR + ∆P |  |  |
| Low drive (ref: moderate drive) | 1.36 (0.68-2.72) | 0.049 |
| High drive (ref: moderate drive) | 2.62 (1.20-5.73) |  |
| Respiratory drive class + Study Arm + Non-pulmonary SOFA + PaO_2_/FiO_2_ + VR + V_T_/PBW + P_L_,insavg + \|P_L_,expavg\| |  |  |
| Low drive (ref: moderate drive) | 1.49 (0.74-3.00) | 0.046 |
| High drive (ref: moderate drive) | 2.69 (1.23-5.90) |  |
| Respiratory drive class + Study Arm + SOFA + ∆P + log-angiopoietin-2 |  |  |
| Low drive (ref: moderate drive) | 1.27 (0.63-2.57) | 0.04 |
| High drive (ref: moderate drive) | 2.63 (1.21-5.73) |  |
| Respiratory drive class + Study Arm + SOFA + ∆P + log-interleukin-6 |  |  |
| Low drive (ref: moderate drive) | 1.34 (0.68-2.66) | 0.03 |
| High drive (ref: moderate drive) | 2.77 (1.27-6.03) |  |
| Respiratory drive class + Study Arm + SOFA + ∆P + log-interleukin-8 |  |  |
| Low drive (ref: moderate drive) | 1.33 (0.68-2.63) | 0.03 |
| High drive (ref: moderate drive) | 2.85 (1.30-6.27) |  |
| Systemic corticosteroid use did not differ by respiratory drive class, was not associated with survival, and therefore was not included in models.  *Abbreviations*: ∆P, airway driving pressure; P_L_,insavg, average end-inspiratory transpulmonary pressure through study day 3; \|P_L_,expavg\|, average of the absolute value of end-expiratory transpulmonary pressure through study day 3; SOFA, sequential organ failure assessment score; V_T_/PBW, tidal volume in mL/kg predicted body weight; VR, ventilatory ratio. | | |

| **Table E7. Association of Respiratory Drive (P_ES_0.1) with Ventilator-Free Days: Zero-Inflated Poisson Models** | | |
| --- | --- | --- |
| **Model Specification** | **Incidence Rate Ratio (95% CI)** | **p-value** |
| Respiratory drive class only |  |  |
| Low drive (ref: moderate drive) | 0.96 (0.86-1.06) | 0.09 |
| High drive (ref: moderate drive) | 1.14 (0.98-1.33) |  |
| Respiratory drive class + Study Arm + SOFA + ∆P |  |  |
| Low drive (ref: moderate drive) | 0.94 (0.84-1.06) | 0.04 |
| High drive (ref: moderate drive) | 1.18 (0.99-1.40) |  |
| Respiratory drive class + Study Arm + non-pulmonary SOFA + PaO_2_/FiO_2_ + VR + ∆P |  |  |
| Low drive (ref: moderate drive) | 0.91 (0.81-1.02) | 0.02 |
| High drive (ref: moderate drive) | 1.15 (0.97-1.37) |  |
| Respiratory drive class + Study Arm + Non-pulmonary SOFA + PaO_2_/FiO_2_ + VR + V_T_/PBW + P_L_,insavg + \|P_L_,expavg\| |  |  |
| Low drive (ref: moderate drive) | 0.97 (0.87-1.09) | 0.03 |
| High drive (ref: moderate drive) | 1.20 (1.02-1.42) |  |
| *Abbreviations*: VR, ventilatory ratio; ∆P, airway driving pressure; SOFA, sequential organ failure assessment score; V_T_/PBW, tidal volume in mL/kg predicted body weight; P_L_,insavg, average end-inspiratory transpulmonary pressure through study day 3; \|P_L_,expavg\|, average of the absolute value of end-expiratory transpulmonary pressure through study day 3. | | |
